# Which specimens from a museum collection will yield DNA barcodes? A time series study of spiders in alcohol

**DOI:** 10.3897/zookeys.365.5787

**Published:** 2013-12-30

**Authors:** Jeremy A. Miller, Kevin K. Beentjes, Peter van Helsdingen, Steven IJland

**Affiliations:** 1 Naturalis Biodiversity Center, Postbus 9517, 2300 RA Leiden, the Netherlands; 2 Department of Entomology, California Academy of Sciences, 55 Music Concourse Drive, Golden Gate Park, San Francisco, California 94118, USA; 3 Plazi, Zinggstrasse 16, Bern, Switzerland; 4 European Invertebrate Survey – Nederland, Postbus 9517, 2300 RA Leiden, the Netherlands; 5 Gabriel Metzustraat 1, 2316 AJ Leiden, the Netherlands

**Keywords:** DNA extraction, DNA concentration, DNA preservation, DNA degradation, independent contrasts, museum

## Abstract

We report initial results from an ongoing effort to build a library of DNA barcode sequences for Dutch spiders and investigate the utility of museum collections as a source of specimens for barcoding spiders. Source material for the library comes from a combination of specimens freshly collected in the field specifically for this project and museum specimens collected in the past. For the museum specimens, we focus on 31 species that have been frequently collected over the past several decades. A series of progressively older specimens representing these 31 species were selected for DNA barcoding. Based on the pattern of sequencing successes and failures, we find that smaller-bodied species expire before larger-bodied species as tissue sources for single-PCR standard DNA barcoding. Body size and age of oldest successful DNA barcode are significantly correlated after factoring out phylogenetic effects using independent contrasts analysis. We found some evidence that extracted DNA concentration is correlated with body size and inversely correlated with time since collection, but these relationships are neither strong nor consistent. DNA was extracted from all specimens using standard destructive techniques involving the removal and grinding of tissue. A subset of specimens was selected to evaluate nondestructive extraction. Nondestructive extractions significantly extended the DNA barcoding shelf life of museum specimens, especially small-bodied species, and yielded higher DNA concentrations compared to destructive extractions. All primary data are publically available through a Dryad archive and the Barcode of Life database.

## Introduction

The DNA barcoding enterprise has demonstrated its utility for contributing to studies of both well-known and poorly-known taxonomic communities. Studies of diverse tropical arthropods often include many species without formal names (e.g. Smith et al. 2005, Janzen et al. 2009). DNA barcode sequences in conjunction with morphological data are a potent combination for a wide range of biodiversity applications (Dayrat 2005, Will et al. 2005, Goldstein and DeSalle 2011, Riedel et al. 2013). The focus of this research is to develop a DNA barcode library for a well-known fauna: Dutch spiders. The list of spider species recorded from the Netherlands, which stands as of this writing at 644, has been extensively documented and periodically updated through the Fauna Europaea database (Helsdingen 1999, 2013). The specimens necessary to build such a library come from collections, either fresh material or natural history museums. The national natural history collection for the Netherlands is curated at the Naturalis Biodiversity Center. We investigated how a variety of factors (time since collection, body size, phylogenetic distance) influence the success of DNA barcode sequencing. Our goal is to characterize which specimens in the collection are or are not likely to yield a successful DNA barcode sequence, and to use this knowledge to efficiently build a barcode library based on a combination of fresh and museum specimens.

A collection like Naturalis makes large numbers of spider specimens accessible for research, including many rare species. Traditional natural history museums like Naturalis store collections in cool, dark environments to keep specimens preserved over long periods of time. However, these conditions are inadequate to completely prevent degradation of specimen DNA. Spider collections are typically preserved in 70-80% ethanol. At these concentrations, ethanol has oxidative and hydrolytic effects that can degrade DNA over time (Vink et al. 2005). DNA degradation eventually proceeds to the point that the standard animal DNA barcode locus, a ~650 base pair region of the mitochondrial cytochrome *c* oxidase subunit I gene (COI), fails to amplify using basic protocols. It may still be possible to sequence part or all of the DNA barcode region by amplifying a series of short sections and reassembling them (Van Houdt et al. 2010, Andersen and Mills 2012, Zuccon et al. 2012), but this approach requires a substantial increase in time and resources devoted per specimen.

Freshly collected specimens present fewer technical obstacles to successful DNA barcode sequencing. Obtaining and processing samples requires some time and effort. Sample contents are influenced by a wide range of factors, including weather, season, and collecting methodology. So perhaps beyond some common species, one cannot predict with certainty which species will be represented in the samples.

Fresh and museum collections have complementary strengths and weaknesses when it comes to the efficient development of a DNA barcode library. Initially, field work generates fresh specimens of many species in need of barcoding. As the DNA barcode library grows, it eventually becomes increasingly difficult to find fresh specimens of species that have not been barcoded previously. This may be true even while the number of barcoded species is substantially lower than the number of species known from the Netherlands. This may be the time to turn to the museum collection and specifically target species that have eluded current field work. However, natural history museums are a resource for the global research community and activities that can damage museum specimens, including DNA extraction, should be undertaken with consideration that the anticipated research value will outweigh any specimen degradation. To this end, we have investigated barcode sequencing success rates as a function of years since collection, considering both destructive and nondestructive DNA extraction methods. Species representing a variety of spider lineages and a range of body sizes were included.

## Methods

### Fresh collections

Spiders were collected from several locations in the Netherlands. Collecting methods included beating or sweeping vegetation, sifting leaf litter, and hand collecting. 70% Ethanol was used as a preservative. Samples were kept at -20 °C when not being worked on. Specimens were identified by taxonomic experts on the Dutch spider fauna and exemplars were selected for DNA barcoding.

## Museum collection

31 frequently collected species were selected (Figure 2). For the 1990s and 2000s, 1–4 specimens of each species were selected per decade, and 1–2 specimens per decade were selected as available going back to 1950. This was supplemented with 1-3 fresh or museum specimens from 2010–2012. Specimens collected using pitfall traps were avoided because the preservative formalin, commonly used in pitfalls, damages DNA (Gurdebeke and Maelfait 2002). However, historical specimen data labels may not always indicate when specimens were collected using formalin pitfalls. All 31 time series species yielded DNA barcode sequences for at least some specimens, indicating that sequencing failures could not be attributed to a lack of primer specificity.

The Naturalis spider collection has been kept (along with most of the Naturalis collection) in a 60 m collection tower since 1998. Conditions are controlled and monitored, with temperature maintained between 17–18 °C and relative humidity 50–55%. We have been unable to find data on conditions prior to the move to the tower. Specimens are kept in cotton-stoppered glass vials; up to several dozen vials are kept together submerged in 70% ethanol within a larger jar. This is intended to keep ethanol concentration stable.

### DNA barcode sequencing

Initial source tissue for both fresh and museum specimens was a single leg, removed from the specimen and ground using a sterile blade in a 1.2 ml eppendorf tube, then incubated for three hours in lysis buffer with proteinase K. For second round extractions from selected museum specimens, DNA was extracted by placing the entire specimen (minus one leg consumed by destructive extraction) directly (without grinding) in lysis buffer with proteinase K for the three hour incubation step. After incubation, the specimen was returned to ethanol and the extraction continued using the lysis buffer solution. This caused negligible to slight further damage to the specimen (Rowley et al. 2007, Paquin and Vink 2009). These two methods are referred to in this paper as destructive and nondestructive extraction, respectively. Some of the larger species (*Araneus quadratus* Clerck, 1757, *Tegenaria atrica* C. L. Koch, 1843, *Dolomedes plantarius* Clerck, 1757) could not be fit into the extraction tubes without damage and were excluded from the nondestructive extraction portion of the study.

Extractions proceeded using the Thermo Scientific KingFisher Flex magnetic bead extraction robot at the Naturalis Biodiversity Center DNA barcoding facility using the Macherey-Nagel NucleoMag 96 Tissue kit. To obtain the standard animal DNA barcode fragment of the mitochondrial COI gene (Hebert et al. 2003), PCR was performed using the primers LCO1490 (5’-GGTCAACAAATCATAAAGATATTGG-3’) (Folmer et al. 1994) and Chelicerate Reverse 2 (5’-GGATGGCCAAAAAATCAAAATAAATG-3’) (Barrett and Hebert 2005). PCR reactions contained 18.75 µl mQ, 2.5 µl 10 × PCR buffer CL, 1.0 µl 25 mM of each primer, 0.5 µl 2.5 mM dNTPs and 0.25 µl 5U Qiagen Taq. PCR was performed using an initial denaturation step of 180 s at 94 °C, followed by 40 cycles of 15 s at 94 °C, 30 s at 50 °C and 40 s at 72 °C, and finishing with a final extension of 300 s at 72 °C and pause at 12 °C. Sequencing was performed by Macrogen (http://www.macrogen.com) or BaseClear (http://www.baseclear.com/). For all barcoded specimens, sequences, images, and collection data were uploaded to the Barcode of Life Data Systems (BOLD; http://www.boldsystems.org/) in the project NLARA “Araneae of the Netherlands”. DNA concentration was assessed using 1.5 µl samples of genomic DNA extract run through a NanoDrop ND-1000 Spectrophotometer (www.nanodrop.com/).

### Correlates of sequencing success and failure

We used independent contrasts (Felsenstein 1985, Garland et al. 1992) to investigate species body size and phylogenetic distance as factors that might explain the oldest successful sequence from the 31 frequently collected species. The independent contrasts method factors out the phylogenetic non-independence of species so that correlations between two continuous variables can be validly tested on a collection of species. Each species was scored for body size and years since collection for the oldest successful DNA barcode sequence. Male and female body sizes were taken from the literature (Roberts 1985, 1987, Nentwig et al. 2013) and averaged. A single exemplar sequence representing each focal species was taken from the freshest available specimen. We generated a Neighbour-Joining tree in DAMBE (Xia and Xie 2001; F84 model, 10 000 random addition steps). We used the PDAP package in Mesquite (Midford et al. 2010, Maddison and Maddison 2011) to perform independent contrasts analysis. Other statistical analyses (log_10_ transformation, Pearson’s r correlation, ANOVA and *χ*^2^) were performed using PAST (Hammer et al. 2001).

The amount of tissue taken from each specimen for destructive DNA extraction was not quantified or controlled for and was substantially different among the species in the study. We therefore investigated the role of DNA concentration. We looked for a relationship between 1) body size and 2) years since collection against DNA concentration (ng/µl) and DNA barcode sequencing success rates for specimens included in the time series study based on both destructive and nondestructive extraction.

Recent collections covered a broader set of species than the time series study. Tree-based methods like independent contrasts are not applicable to this dataset because species that failed to produce a DNA barcode sequence could not be included in the tree. We searched the BOLD databases for sequences to represent these species, but a substantial number (9 of 14) are currently not available. Body size was calculated as for the time series species.

## Data resources

All occurrence data for specimens included in this study are available as part of a Dryad (http://datadryad.org/) data package (doi: 10.5061/dryad.4bq08). Occurrence data are presented as a tab delimited text file with Darwin Core fields (http://darwincore.googlecode.com/svn/trunk/terms/index.htm), plus custom fields for recording destructive and nondestructive sequencing success, DNA sequences, DNA concentration data, and hyperlinks to records on BOLD (http://www.boldsystems.org/). Also included in the Dryad data package is a KML file that can be opened using Google Earth (http://earth.google.com/) to display an interactive map plotting Dutch spider specimens included in this study. Click on placemarks to reveal specimen data and, where available, a hyperlink to sequence data for that specimen on BOLD (http://www.boldsystems.org/). The Dryad data package also includes all sequence data for this study in fasta format, two Nexus files generated using Mesquite (Maddison and Maddison 2011) for the independent contrasts analyses, and Appendix - Figure S1 illustrating correlations based on independent contrasts analyses.

## Results

**Figure 1.**
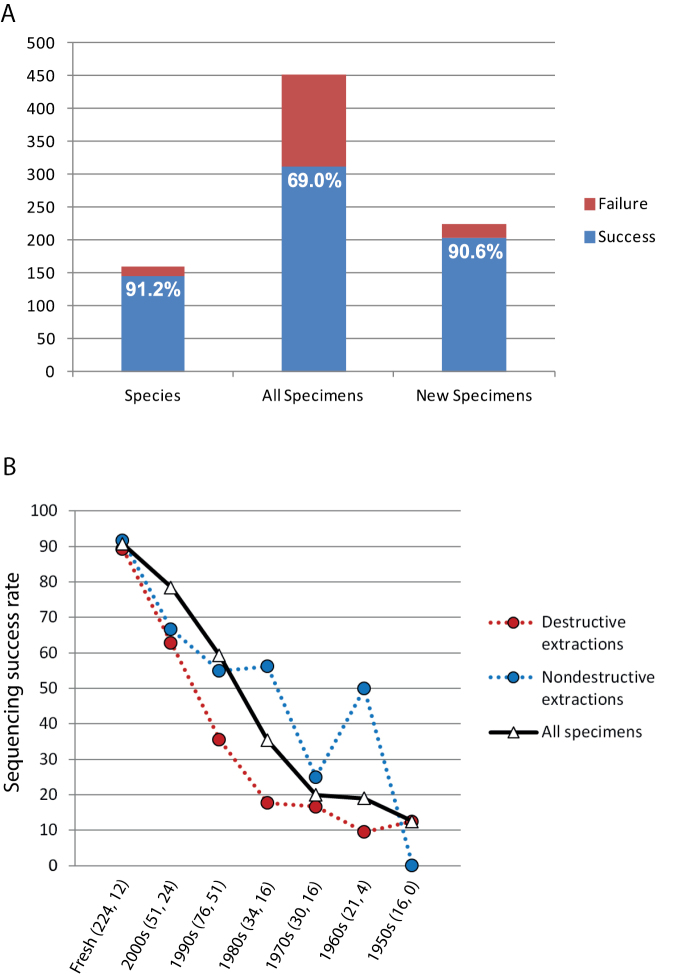
**A** Sequencing success profile for specimens included in this study. Data are species attempted, all specimens in the study including the time series, and fresh specimens collected in 2010 or later. Success expressed as a percentage appears on the blue (success) portion of each bar **B** Sequencing success rates for fresh (collected 2010 or later) and older specimens grouped by decade. Data given for all extractions regardless of method, and also partitioned into destructive and nondestructive extraction methods. Total number of specimens attempted and the subset of specimens attempted using nondestructive extraction given in parentheses. Note that the relatively high success rate for nondestructive extractions of specimens from the 1960s is based on two successes out of four attempts.

We obtained DNA barcode sequences for 145 spider species (91.2% of the 159 species attempted) based on 452 fresh and museum specimens (Figure 1A). Sequences ranged from 510 to 658 bp (mean 650.1). The 14 species attempted that failed to yield a DNA barcode were *Clubiona subtilis* L. Koch, 1867 (Clubionidae); *Harpactea hombergi* (Scopoli, 1763) (Dysderidae); *Haplodrassus silvestris* (Blackwall, 1833) (Gnaphosidae); *Cnephalocotes obscurus* (Blackwall, 1834), *Dismodicus elevatus* (C.L. Koch, 1838), *Entelecara congenera* (O. Pickard-Cambridge, 1879), *Erigone dentipalpis* (Wider, 1834), *Gnathonarium dentatum* (Wider, 1834), *Gongylidium rufipes* (Linnaeus, 1758), *Macrargus rufus* (Wider, 1834), *Walckenaeria antica* (Wider, 1834) (Linyphiidae); *Arctosa leopardus* (Sundevall, 1833) (Lycosidae); *Pholcus phalangioides* (Fuesslin, 1775) (Pholcidae); and *Pachygnatha listeri* Sundevall, 1830 (Tetragnathidae).

**Figure 2.**
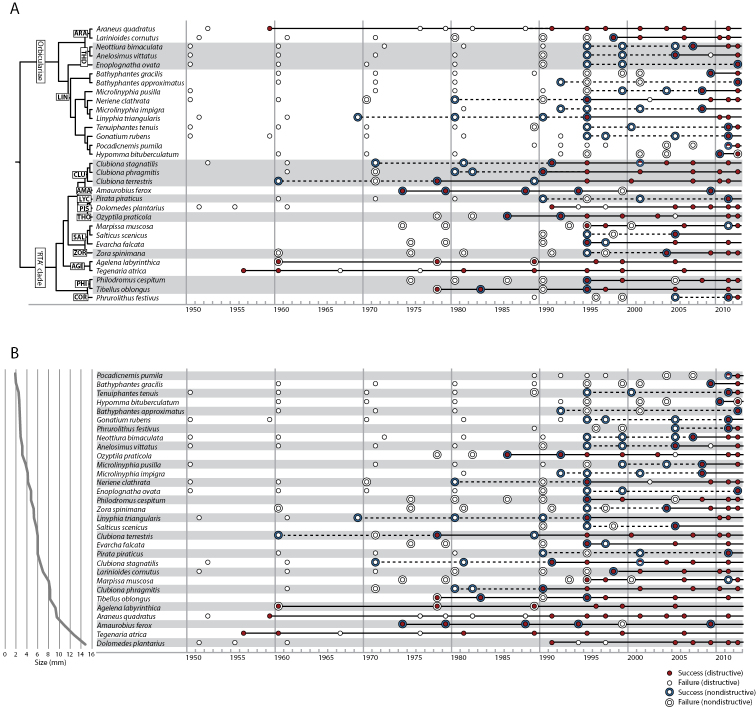
Sequencing success for the time series study of 31 spider species frequently collected in the Netherlands. Data for each species arranged horizontally along a time axis (year of collection). Small circles represent standard destructive extraction; outer circle represents nondestructive extraction. Red small circle and blue outer circle indicate successful sequencing, unfilled circles represent failed attempts; half-filled circle indicates mixed success among multiple specimens for that species and year. Solid horizontal lines extend from the present to the oldest successful DNA barcode based on destructive extraction for each species; where nondestructive extraction yielded successful DNA barcode from older specimens, this is indicated by a dashed line. Data are arranged according to a neighbour joining tree (**A**) or by species body size (**B**). Spider families and major lineages (Orbiculariae and ‘RTA’ clade) are indicated in **A**. **AGE** Agelenidae **AMA** Amaurobiidae **ARA** Araneidae **CLU** Clubionidae **COR** Corinnidae **LIN** Linyphiidae **LYC** Lycosidae **PHI** Philodromidae **PIS** Pisauridae **SAL** Salticidae **THD** Theridiidae **THO** Thomisidae **ZOR** Zoridae.

For fresh specimens (collected 2010 or later), the overall sequencing success rate was 90.6%. For specimens collected between 2000 and 2009, the success rate drops slightly to 78.4%. For specimens collected in the 1990s, sequencing success drops to 59.2%, then to 35.3% for specimens collected in the 1980s, then to around 20% for specimens collected in the 1970s and 1960s, and finally 12.5% for specimens collected in the 1950s (Figures 1, 2).

When genetic distance is accounted for using independent contrasts, we found a significant positive correlation between body size and years since collection for successful DNA barcode sequences (Appendix - Figure S1). Using our protocol and a single long run PCR, the standard DNA barcode sequences can be obtained from larger spider species for a longer period of time compared to smaller spider species. This relationship holds regardless of whether we consider only data from destructive extractions (*R*^2^ = 0.39, *F* (1, 29) = 18.87, *p* = 1.56E-4) or all extractions (*R*^2^ = 0.23, *F* (1, 29) = 8.43, *p* = 6.99E-3) despite the fact that three of the species were too large to include in the nondestructive extraction portion of the study.

**Figure 3.**
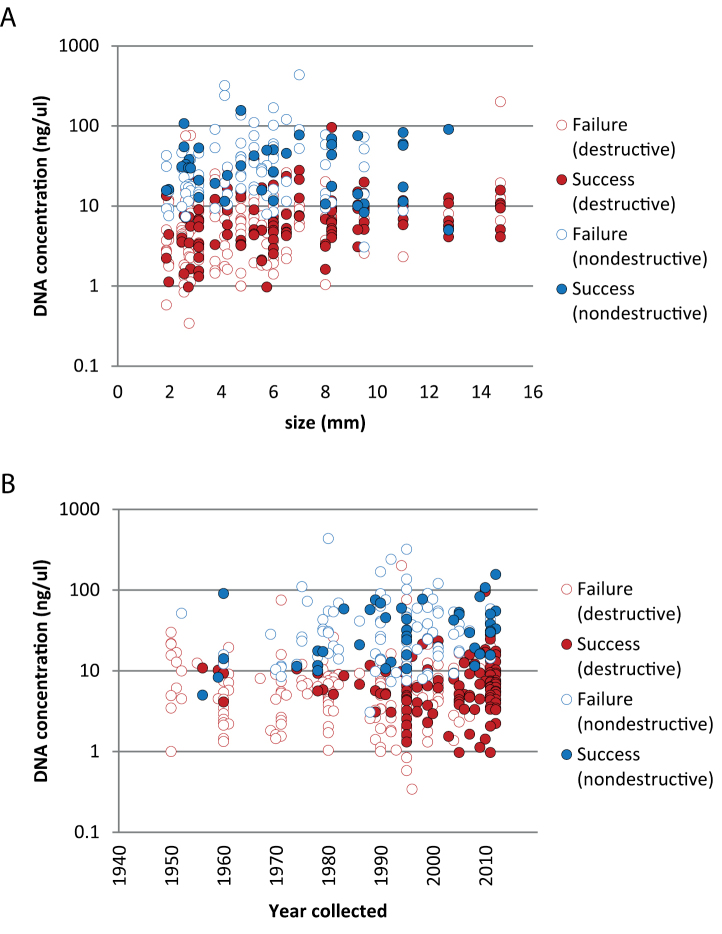
DNA concentration (log_10_ transformed) for specimens in the time series study that yielded or failed to yield a successful DNA barcode sequence arranged by **A** body size **B** year collected. Successes (filled circles) and failures (while circles) partitioned into destructive (red) and nondestructive (blue) DNA extraction methods.

**Figure 4.**
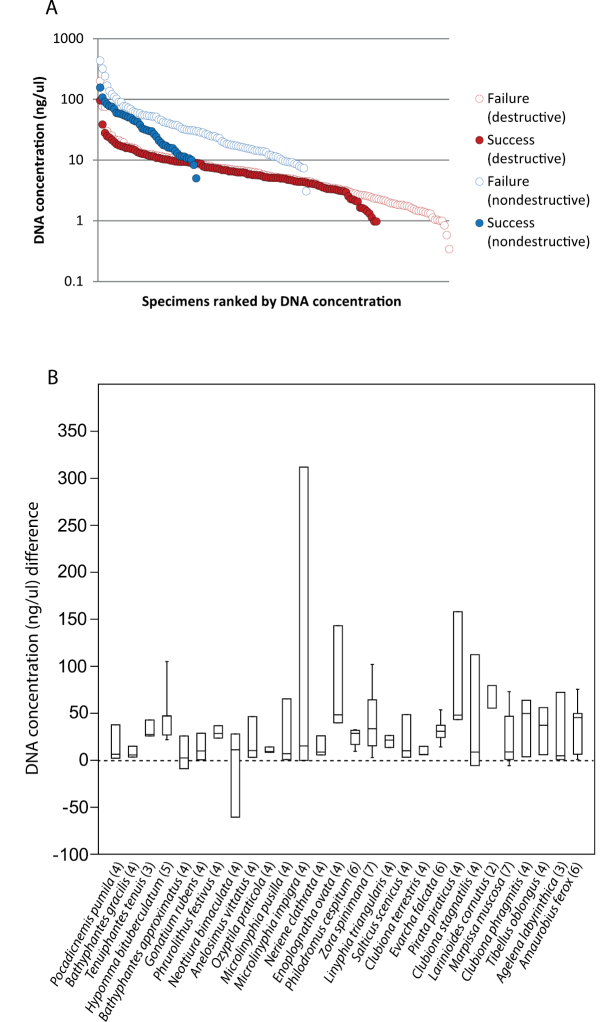
**A** DNA concentration (log_10_ transformed) for specimens in the time series study that yielded or failed to yield a successful DNA barcode sequence ranked by DNA concentration; symbols as in Figure 3 **B** Box plot showing difference in DNA concentration for specimens extracted using both destructive and nondestructive methods; species arranged by size (*Araneus quadratus*, *Tegenaria atrica*, and *Dolomedes plantarius* excluded). Sample size in parentheses, boxes are 25–75% quartiles bisected by the median, whisker lines indicate minimum/maximum values (where *n* > 4).

Body size is correlated with DNA concentration based on data from destructive extractions (*r* (281) = 0.30, *p* = 2.31E-03); this relationship is not evident for the smaller dataset based on non-destructive extractions (*r* (130) = 0.05, *p* = 0.61). Years since collection is correlated with DNA concentration based on data from the non-destructive extractions (*r* (130) = 0.20, *p* = 0.02) but not the destructive extractions (*r* (281) = 0.01, *p* = 0.92). In all cases, the dependent variable was log_10_ transformed. Nondestructive extractions did yield significantly higher concentrations compared to destructive extractions (Figures 3, 4; one-way ANOVA, *p* < 0.05 whether considering only extracts that produced a barcode sequence (*F* (1, 159) = 120.2, *p* = 3.45E-18), extracts that failed (*F* (1, 232) = 184.1, *p* = 295E-28), or all extracts measured (*F* (1, 395) = 305.7, *p* = 4.19E-48). In all cases, concentration values were log_10_ transformed. Note that nondestructive samples all had one leg removed (consumed for destructive samples); we don’t know what effect this might have had on barcoding success since the space left by the removed leg leading to the interior of the prosoma may have facilitated the extraction.

Of 123 samples where both destructive and nondestructive extraction methods were tried, 38 produced successful barcodes using destructive extraction and 85 produced successful barcodes using nondestructive extraction. Of the 38 successful destructive extraction barcodes, 32 (84.2%) were also successful using nondestructive extraction while 6 (15.8%) failed. Of the 85 unsuccessful destructive barcodes, 38 (44.7%) were successful using nondestructive extraction while the remaining 47 failed using both methods. So although nondestructive extraction failed in about 15% of the cases where destructive sampling was successful, nondestructive extraction was significantly better at yielding successful barcode sequences, particularly when destructive extraction failed (*χ*^2^ (2, *N* = 123) = 16.71, *p* = 0.0002).

The combination of destructive and nondestructive extractions extended the DNA barcoding shelf life of the species in our study over destructive extraction alone by an average of 9.3 years. The nondestructive portion of our study was not comprehensive, involving only 123 (44.6%) of the specimens and 28 (90.3%) of the species in the time series study. The oldest successful barcode specimen was on average 6.7 years older for the nondestructive extraction data compared to the destructive extractions. The oldest successful barcode template was from a nondestructive extraction in 17 of the 28 species compared (60.7%); the oldest successful barcode template came from a destructive extraction in only 3 of the species (10.7%). However, for one of these species (*Agelena labyrinthica* (Clerck, 1757)) the nondestructive extraction never produced a successful barcode sequence while the destructive extractions were effective for every specimen attempted (*n* = 6) going back to 1960. In *Marpissa muscosa* (Clerck, 1757), destructive extractions were also much more effective than nondestructive extractions (Figure 2).

## Discussion

**Figure 5.**
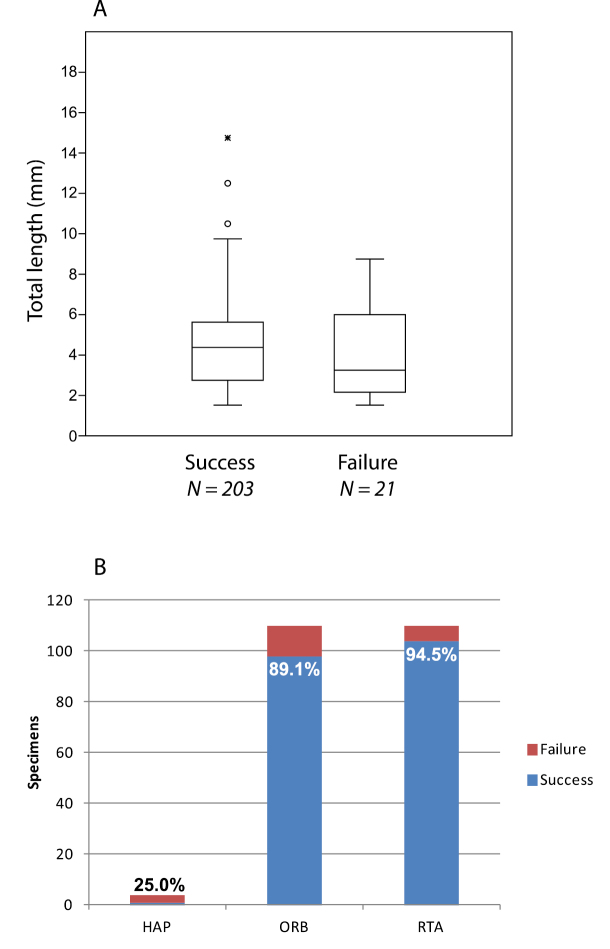
DNA barcode sequencing success for fresh specimens (collected 2010 or later). **A** Specimen body size not significantly different for successful vs. failed DNA barcode sequencing attempts (one-way ANOVA, *F* (1, 216) = 1.45, *p* = 0.230). Boxes are 25–75% quartiles bisected by the median, whisker lines drawn to the largest/smallest data point less than 1.5 times the box height, outliers less than 3 times the box height shown as circles, more than 3 shown as stars. **B** Most of the fresh specimens included in this study belonged to one of two clades: Orbiculariae (ORB) or the ‘RTA’ clade (RTA); only a handful of specimens represented older phylogenetic branches, such as haplogyne (HAP) spiders; no mygalomorph spiders were included; success expressed as a percentage appears on or above each bar. Success rate for Orbiculariae vs. ‘RTA’ clade specimens not significantly different (*χ*^2^ = 2.18, d.f. = 2, *N* = 220, *p* = 0.337).

Failure rates for DNA barcode sequencing rise with time since collection, but body size is a significant factor. For freshly collected specimens overall, body size is not a predictor of sequencing success or failure (Figure 5A). But larger species have a longer DNA barcoding shelf life than smaller species under museum collection conditions, at least using a single pair of primers to amplify the entire ~650 base pair region in one reaction. This may be explained in part by the finding that concentration of extracted DNA is correlated with specimen size and inversely correlated with specimen age, but this relationship is neither strong nor consistently found. The dominant protocol for spider DNA barcoding and other Sanger sequencing involves the removal of tissue from the specimen, typically from one or more legs. Our data suggest that nondestructive extraction techniques can significantly improve the chances of obtaining a DNA barcode sequence. Considering only the commonly applied destructive extraction technique, small spiders are useful for only a few years while those with a body size of around 3 mm or more have a modest chance of yielding a barcode sequence for about 20 years after collection. But with judicious application of nondestructive extraction, spiders from museum collections with a body length of 4 mm or less have a modest chance of yielding a DNA barcode sequence from a single PCR reaction for about 15 years since collection while spiders above this size can yield barcode sequences for a considerably longer time. For some of the larger species, we did not include specimens old enough to fail to produce DNA barcodes, so their real shelf life may be even longer than indicated here (Figure 2B).

All of the species in the time series study and nearly all the fresh specimens attempted belong to two major sister clades: the Orbiculariae (orb web weavers and their descendents) and the ‘RTA’ clade (so named for the synapomorphic retrolateral tibial apophysis of the male pedipalp; Coddington and Levi 1991). Together, these clades account for about 83% of described spider diversity (Platnick 2013). Recent field work found very few representatives of spider lineages that branched off before the origin of the Orbiculariae+’RTA’ clade (e.g. Haplogynae and other early branching araneomorphs, or Mygalomorphae, which account for only 20 and 3 of the 644 recorded Dutch spider species respectively; Figure 5B). So results reported here may not be generalizable beyond this major spider lineage. Our data indicate no difference in failure rate for Orbiculariae compared to the ‘RTA’ clade (*χ*^2^ = (2, *N* = 220) = 2.18, *p* = 0.34; Figure 5B).

We found no differences in sequencing success rate by lineage. It may yet be that changes in chemistry (e.g. DNAase, PCR inhibitors), primer binding site sequences, or other heritable characteristics might make some spider lineages more resistant to sequencing than others.

Several recent studies have investigated the relationship between specimen age and DNA barcode sequencing success for museum collections (Van Houdt et al. 2010, Andersen and Mills 2012, Zuccon et al. 2012). These studies include PCR reactions targeting short portions of the DNA barcode region as a way of compensating for the DNA degradation that comes with time. With field collection ongoing, we do not yet know which species available in the museum collection might elude contemporary field work. As field work becomes increasingly inefficient at producing fresh specimens of unbarcoded species, the museum collection may become the only readily available source for certain species. Based on what we have learned through this study about body size and specimen age, we will be able to predict whether standard protocols are likely to produce a successful DNA barcode sequence, or if more refined and targeted methods including PCRs targeting one or more sub-regions of the DNA barcode, should be employed. The success of nondestructive extraction demonstrated here coupled with the need to preserve museum specimens for a variety of research purposes bodes well for museum collections as a source of material for spider barcode libraries, and perhaps other alcohol collections as well.

### DNA barcoding spiders in Europe

The initiative to create a library of DNA barcode sequences for Dutch spiders occurs in a broader context. Research teams in several European countries are involved in similar national projects (see http://www.araneae.unibe.ch/barcoding/content/15/Barcoding-of-European-spiders). The synergies anticipated from multiple libraries across Europe and beyond are exciting. As these libraries mature, they will become a reference not only for taxonomic identification, but for assessing intraspecific variation across the region. As barcode sequence data are independent of the morphological characters traditionally used to establish and subsequently recognize species, they will provide a check of species concepts as applied internationally. We may find that some species considered widespread exhibit sufficient sequence variation and geographical structure to warrant further study, or discover a lack of variation in different nominal species that could indicate these species are in fact one. Of the nearly 4 900 spider species recorded from Europe, more than 2 000 are known from only one country (Helsdingen 2013). It may well be that some portion of this national endemism is an artifact.

The development of a DNA barcode library of European spiders is too large a task for any one research group. Data standards and a community data repository facilitate the reuse and reevaluation of DNA barcode data generated by independent labs (Ratnasingham and Hebert 2007). The increasing adoption by the scientific community of data standards and online resources for data aggregation strengthens both cooperative and adversarial (i.e., independent repeatability) aspects of biodiversity research, contributing to both productivity and rigor (Johnson 2011). As the data become aggregated, inconsistencies will be revealed suggesting possible errors that should be investigated and corrected using an approach that integrates data from all available sources including morphology (Dayrat 2005, Will et al. 2005, Goldstein and DeSalle 2011, Riedel et al. 2013).

### Beyond barcoding

In recent years, cost curves for next generation DNA sequencing technologies (NGS) have been falling. As time goes on, it seems inevitable that NGS will become increasingly competitive with traditional Sanger sequencing. NGS approaches are less dependent on long intact DNA fragments compared to the long run Sanger barcoding demonstrated here (Ekblom and Galindo 2011, Lemmon et al. 2012). This suggests that spider collections such as the one at Naturalis may be even richer as a source of data for NGS studies than we found using traditional sequencing.

## Appendix

Electronic supplementary documents. (doi: 10.3897/zookeys.365.5787.app) File format: WinZip Archive. (zip).

**Explanation note:** Archive contents:

Figure S1.doc – Correlations based on independent contrasts.

Milleretal2013DutchSpiderBarcodeDwC.txt – Occurrence data for all specimens in the study.

Milleretal2013DutchSpiderBarcode.kml – Occurrence data in KML (keyhole markup language.

Milleretal2013DutchSpiderBarcode.fasta – All sequence data for this study in fasta format.

Milleretal2013DutchSpiderBarcodeDestructiveExtractions.nex – Nexus files generated using Mesquite (http://mesquiteproject.org).

Milleretal2013DutchSpiderBarcodeAllExtractions.nex – Nexus files generated using Mesquite (http://mesquiteproject.org).
