# Supplementary material for: Which specimens from a museum collection will yield DNA barcodes? A time series study of spiders in alcohol
Source: Zookeys. 2013 Dec 30;(365):245–61. doi: 10.3897/zookeys.365.5787 (PMC3890681; doi:10.3897/zookeys.365.5787)
Supplement: Supplementary file 1 — Electronic supplementary documents. (doi: 10.3897/zookeys.365.5787.app) File format: WinZip Archive. (zip). [file ZooKeys-365-245-s001.zip › Figure S1.docx]

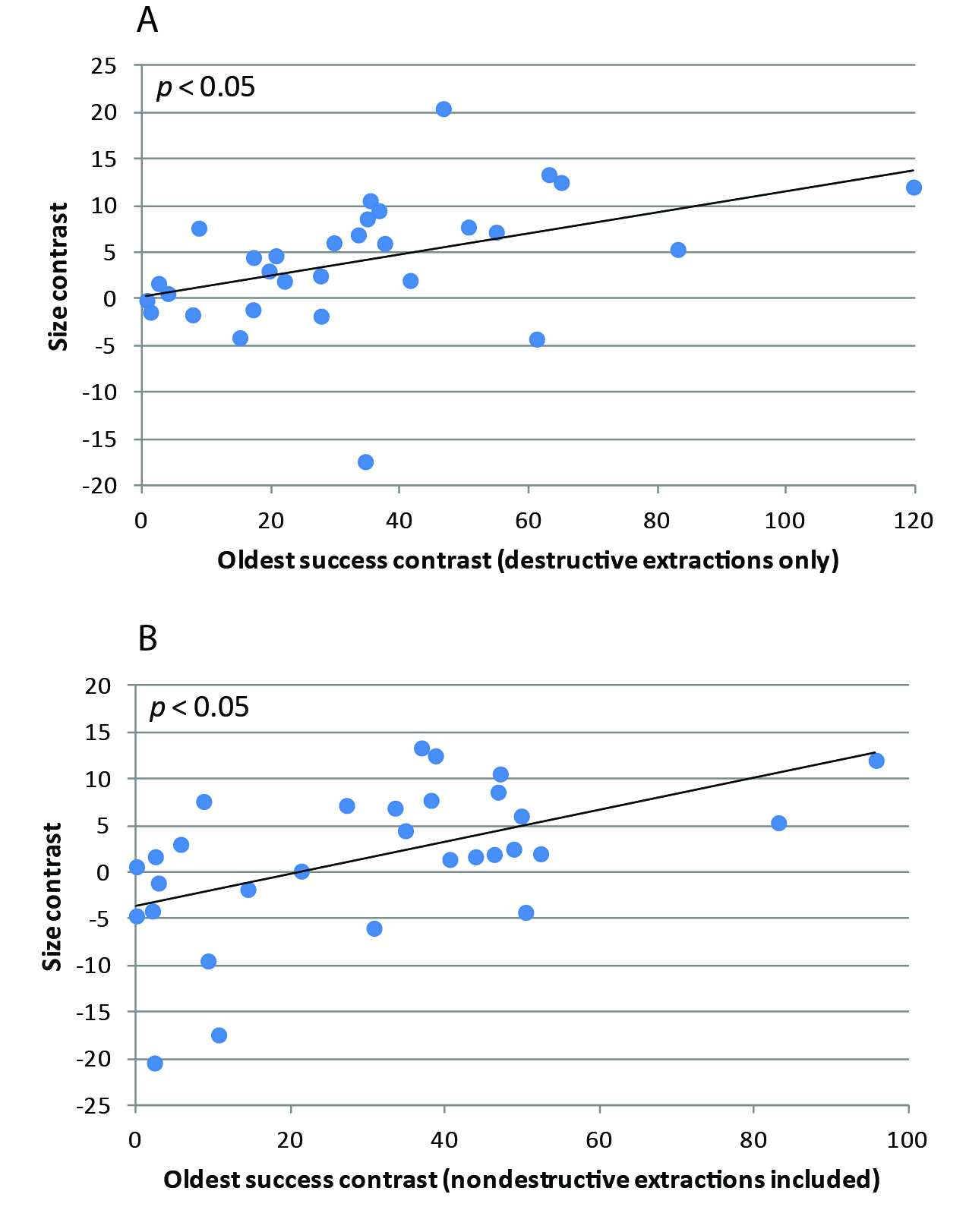


**Figure S1.** Independent contrasts analysis finds a significant correlation between body size and oldest DNA barcoding success after factoring out genetic distance among species. Data from (**A**) destructive extractions only and (**B**) nondestructive extractions included. For both datasets, two-tailed p-value < 0.05.
